# A Role of Stress Sensor Nrf2 in Stimulating Thermogenesis and Energy Expenditure

**DOI:** 10.3390/biomedicines9091196

**Published:** 2021-09-10

**Authors:** Seo-Hyuk Chang, Jeong-Soo Lee, Ui Jeong Yun, Kye Won Park

**Affiliations:** 1 Department of Food Science and Biotechnology, Sungkyunkwan University, Suwon 16419, Korea; adc153@naver.com; 2 Disease Target Structure Research Center, KRIBB, Daejeon 34141, Korea; jeongsoo@kribb.re.kr; 3 Dementia DTC R&D Convergence Program, KIST, Seoul 02792, Korea

**Keywords:** Nrf2, uncoupling, thermogenesis, energy expenditure, obesity, metabolic diseases

## Abstract

During chronic cold stress, thermogenic adipocytes generate heat through uncoupling of mitochondrial respiration from ATP synthesis. Recent discovery of various dietary phytochemicals, endogenous metabolites, synthetic compounds, and their molecular targets for stimulating thermogenesis has provided promising strategies to treat or prevent obesity and its associated metabolic diseases. Nuclear factor E2 p45-related factor 2 (Nrf2) is a stress response protein that plays an important role in obesity and metabolisms. However, both Nrf2 activation and Nrf2 inhibition can suppress obesity and metabolic diseases. Here, we summarized and discussed conflicting findings of Nrf2 activities accounting for part of the variance in thermogenesis and energy metabolism. We also discussed the utility of Nrf2-activating mechanisms for their potential applications in stimulating energy expenditure to prevent obesity and improve metabolic deficits.

## 1. Introduction

In response to external stimuli, physiological signals are required to activate thermogenic effectors, leading to increased thermogenic respiration. Maintaining thermal homeostasis is critical for mammals to survive in cold environments during evolution [1]. During cold exposure, body temperature is maintained by generating heat through contracting muscle involuntarily (shivering thermogenesis) and increasing non-shivering thermogenesis in adipose tissues [2,3]. Brown adipose tissue (BAT) is a specialized organ for adaptive thermogenesis with high expression of uncoupling protein 1 (Ucp1) in mitochondria [4,5]. Due to the catabolizing effect of lipid and glucose stored in BAT by uncoupling, BAT has been considered as a therapeutic strategy for improving metabolic diseases and obesity [6,7].

BAT as a major organ for thermogenesis contains high mitochondrial contents which display elevated levels of reactive oxygen species (ROS) during thermogenesis [8]. Moreover, thiol redox state of BAT is shifting toward more pro-oxidant conditions during cold exposure by generating mitochondrial ROS [9,10,11]. Under these oxidative stress conditions, Nrf2, a master regulator of anti-oxidant response, is activated to remove excess ROS [12,13]. Several studies have shown Nrf2-induced effects on energy expenditure in mouse models either by inhibiting or activating Nrf2 [14,15,16,17,18]. However, Nrf2 activation or suppression exhibits conflicting results in published literature concerning its effects against obesity and metabolic dysregulations. To resolve this discrepancy, we reviewed recent findings regarding the Nrf2 activity on thermogenesis and energy metabolism and further discussed its activation for potential applications in preventing obesity and metabolic diseases.

## 2. Stress Inducible Nrf2 Responds to Cellular Stress

The transcription factor nuclear factor E2 p45-related factor 2 (Nrf2) is cloned for its ability to bind the NF-E2/AP repeat in the promoter of the β-globin gene [19]. It is ubiquitously expressed and dispensable for mouse development [20]. Nrf2 is a major regulator responsible for protection against oxidative stress by controlling the expression of antioxidant genes [21]. Under basal conditions, Nrf2 is maintained at low levels through cytoplasmic retention by Kelch-like ECH-associated protein 1 (Keap1) and constitutive degradation by the ubiquitin-proteasome system in the cytoplasm [22].

**Figure 1:**
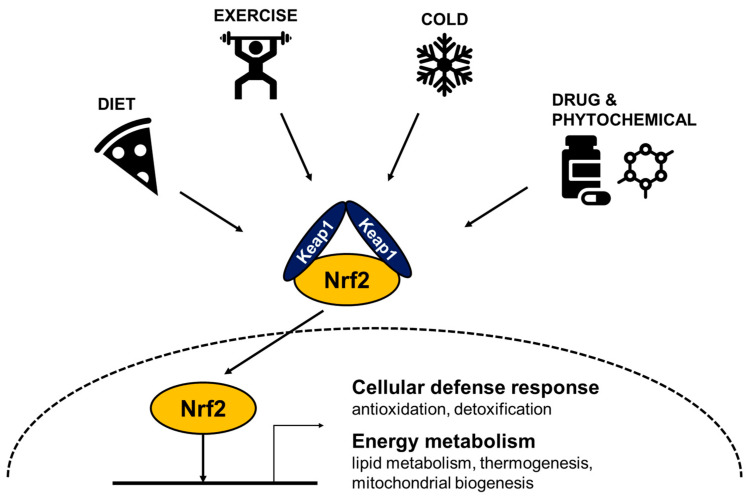
Cellular stresses activate Nrf2 to induce defense systems, reduce body weight, and increase energy expenditure. Schematic diagram of Nrf2-induced responses against various cellular stresses. Cold exposure, physical exercise, food intake, and xenobiotic chemicals activate Nrf2 to transcribe its target genes involved in antioxidation, detoxification, mitochondrial biogenesis, and energy expenditure. ROS can be produced by cold, exercise, high energy diets, and chemical activators that can disrupt Kelch-like ECH-associated protein 1 (Keap1) and Nrf2 interaction to increase Nrf2 abundance and subsequent nuclear translocation of Nrf2. In the nucleus, Nrf2 forms a heterodimer with a small Maf protein and binds to antioxidant response element (ARE) in the promoter region to drive transcription of antioxidative genes. Its target genes include glutamate-cysteine ligase catalytic subunit (GCLC), glutathione reductase, ABCC-family efflux transporter genes, NAD(P)H quinone oxidoreductase1 (NQO1), heme oxygenase1 (HO1), and possibly mitochondrial genes including nuclear respiratory factor 1 (Nrf1), Peroxisome proliferator-activated receptor-gamma coactivator-1a (Pgc1α), and uncoupling protein 1 (Ucp1).

ROS can be produced by various cellular stresses including cold, exercise, high energy diets, and chemical activators that activate Nrf2 to regulate antioxidation, detoxification, mitochondrial biogenesis, and energy expenditure (Figure 1). In response to oxidative stress, Nrf2 dissociates from Keap1 and translocates to the nucleus [23]. In the nucleus, Nrf2 binds to the antioxidant response element (ARE), a cis-acting sequence found in the 5′-flanking region of the genes involved in antioxidant responses. Target genes include catalytic and modulator subunits of glutamate-cysteine ligase and glutathione reductase which are enzymes involved in the synthesis and reduction of glutathione (GSH) from oxidized GSSG [24,25]. Nrf2 also mediates the transcriptional activation of detoxifying enzymes including cytochrome P450-dependent oxygenases, NAD(P)H quinone oxidoreductase1 (Nqo1), glutathione S-transferase (GST), and heme oxygenase1 (Ho1) [26,27,28]. Nqo1 detoxifies quinones to prevent the generation of reactive semiquinones and hydrogen peroxide. GST conjugates hydrophobic electrophiles and lipid hydroperoxides with gluthathione for their excretion and Ho1 catalyzes heme catabolism. In addition, drug transporters involved in drug efflux and anti-apoptotic proteins for cell survival are also regulated by Nrf2 [23,24,29]. As ubiquitous Nrf2 plays a critical role in cell protection from oxidative stress, it has been implicated in various human diseases including cancer, respiratory, neurodegenerative, and metabolic diseases [30].

### 2.1. Oxidative Stress

Since a small amount of ROS can cause harmful effects on cellular functions [31], cells have developed defense mechanisms against oxidative stress using various enzymatic and nonenzymatic mechanisms [32]. However, when the production of ROS overwhelms the cellular anti-oxidant ability, it causes damage to cellular proteins, lipids, and DNA [33]. In addition to disturbing redox balance by increasing oxidized macromolecules, ROS can elicit oxidative stress by decreasing redox buffering capacity of GSH, a non-protein antioxidant species. Since Nrf2 is activated during oxidative stress to provide cellular protective mechanisms, it seems practical to activate Nrf2-mediated antioxidant pathway to remove injurious oxidative stress and improve pathological conditions.

### 2.2. Xenobiotic, Phytochemical and Electrophilic Stress

Xenobiotic electrophiles such as naphthoquinone, crotonaldehyde, methylmercury, acrylamide, and benzoquinone cause extensive and non-selective modification of cellular proteins associated with cytotoxicity and cytoprotection [34,35]. Endogenously produced nitrated oleic acid, 4-8-nitroguanosine 3′,5′-cyclic monophosphate (8-nitrocGMP), estrogen quinone, acrolein, hydroxynonenal, and 15-deoxy-delta(12,14)-prostaglandin J_2_ are expected to activate Nrf2 through chemical modifications on thiols of Keap1 [36,37,38].

Various phytochemicals derived from edible or medicinal plants, artificial synthetic chemicals, vitamins, and endogenous metabolites can function as Nrf2 activators. Phytochemicals, sulforaphane from broccoli sprouts, silymarin and silibinin from *Silybum marianum* (milk thistle), sesamol from sesame seeds, epigallocatechin gallate (EGCG) from green tea, quercetin from vegetables and fruits, and curcumin from rhizome of *Curcuma longa* (turmeric) are naturally occurring antioxidants [39]. Other phytochemicals including luteolin, apigenin, myricetin, rutin, hesperetin, naringenin, daidzein, genistein, butein, and resveratrol have also been shown to possess antioxidative activities [39]. Synthetic BHA (tert-butyl-4-hydroxyanisole), BHT (3,5-di-tert-butyl-4-hydroxytoluene), and tBHQ (tert-butylhydroquinone), vitamin E, tocopherols, and tocotrienols also exhibit antioxidation effects [39,40,41]. In addition to chemical protection by phenolic antioxidants, these antioxidants can also provide cellular protection by inducing Nrf2-mediated defensive enzymes and antiapoptotic proteins [39,42]. Synthetic antioxidants, tocopherols, and phytochemicals in cells can disrupt the interaction between Keap1 and Nrf2 to drive ARE-mediated cytoprotective gene transcription.

### 2.3. Metabolic Stress

Excess ROS has been associated with various chronic diseases including obesity, cancer, respiratory, and neurodegenerative diseases. The ROS levels can be managed by endogenous and supplemented antioxidants. Dietary antioxidants including vitamins C and E, carotenoids, and polyphenols can increase the antioxidant system and slow down disease progression. Epidemiologic studies showed negative association between dietary intake of antioxidants such as carotenoids, vitamin C, and vitamin E and incidence of metabolic diseases, asthma, obstructive pulmonary disease, and Crohn’s disease [43,44,45].

ROS production increases as a result of “excess nutrient” [46,47]. Upon excessive calorie intake, ROS production can be above the physiological threshold because a large number of oxidizable substrates converge in the mitochondria, leading to redox imbalance in human and animal models [46,47,48,49,50,51,52].

ROS produced during exercise also activates Nrf2 signaling [53]. Several animal studies have demonstrated that exercise up-regulates either Nrf2 protein expression and phase II enzyme amounts, or enzyme activity in skeletal muscles, kidney and brain, or in combination [54]. Similarly, Wang et al. [55] showed a positive correlation between exercise-induced H_2_O_2_ levels and Nrf2 expression. Furthermore, it has been shown that physical exercise-induced mitochondrial biogenesis in skeletal muscle requires Nrf2 expression [56,57]. It is thus believed that Nrf2 can mediate metabolic effects during physical exercise in addition to its protective roles against oxidative stress.

Mild hypothermia can augment the stress response pathway by activating Nrf2 and inducing expression of its target genes, resulting in resistance to oxidative stress [58]. Cold stress also activates beta-3 adrenergic receptor (β3-AR) in brown adipose tissues to maintain whole-body temperature, probably by stimulating Ucp1 [59,60]. Interestingly, it seems that β3-AR-induced Ucp1 expression and oxygen consumption in diet-induced obese mice are partly dependent on Nrf2 [61]. These findings suggest the existence of β3-AR-Nrf2-Ucp1 axis in controlling thermogenesis and oxidative stress during cold exposure.

Collectively, metabolic stress induced by hypothermia, excess of nutrients, and exercise can increase ROS levels and result in activation of Nrf2 to regulate energy metabolism and prevent ROS-induced cellular damages (Figure 1).

## 3. Mitochondrial ROS Signaling Induces Thermogenesis

Mitochondria are important cellular organelles contributing to the generation of ROS through respiratory chain and other metabolic machineries [62,63]. Increased intracellular ROS causes damage to cellular components and acts in cellular signaling pathways [33,64]. Mitochondrial ROS have also emerged as signaling molecules that mediate thermogenesis in adipocytes [8,65,66,67]. The important role of mitochondrial ROS in thermogenesis has been demonstrated by Boudina and co-workers in an experiment using mice with SOD deleted specifically in adipocytes (AdSOD2KO) [68]. Mitochondrial superoxide dismutase (SOD2) is the primary enzyme functioning for dismutating mitochondrial superoxide to hydrogen peroxide. SOD deletion in adipocytes elevated superoxide levels, enhanced browning in WAT and mitochondrial uncoupling in BAT [68].

It has been shown that acute activation of BAT thermogenesis by applying either cold exposure (4 °C) or β3-adrenergic stimulus is accompanied by elevated mitochondrial ROS levels [65,69]. Consistent with this phenomenon, depletion of mitochondrial ROS using mitochondria-targeting antioxidant MitoQ prior to cold exposure causes hypothermia and reduction of energy expenditure [8,65]. In this study, Chouchani et al. showed that increased BAT mitochondrial ROS supported thermogenesis in a Ucp1-dependent manner as the inhibitory effects of Mito Q in energy expenditure was not observed in Ucp1 KO mice. Similarly, elevated oxygen consumption upon β3-adrenergic stimulation was significantly decreased when limiting thiol oxidation by NAC treatment [65]. Mechanistically, BAT mitochondrial ROS during thermogenesis oxidatively modified Cys253 residue on Ucp1 to increase its sensitivity to adrenergic activation.

The level of glutathione (GSH), an antioxidant against ROS, is negatively correlated with activation of the thermogenic program. Mitochondria isolated from BAT were found to have highly oxidized status (GSH/GSSG ratio) and this GSH pool was even more oxidized in mitochondria from cold-exposed mice [9]. Pharmacological GSH depletion also induced uncoupling respiration in BAT and stimulated white-to-brown conversion in WAT [70,71]. In line with this, forced reduction of GSH level by Gclm deletion in mice protected from HFD-induced excessive weight gain and adipose deposition [72].

Independent of β-adrenergic signaling, succinate a mitochondrial TCA cycle intermediate released from skeletal muscle is selectively accumulated in 4 °C-activated BAT, leading to increased thermogenic respiration [66]. Succinate treatment in brown adipocytes rapidly increased mitochondrial ROS levels and hyperoxidized cysteine residue on peroxiredoxin3 to sulfonic acid. They also found that succinate stimulated brown adipocyte thermogenesis through succinate dehydrogenase (SDH) mediated oxidation activity. Therefore, redox modification of mitochondrial metabolic proteins including Ucp1 and peroxiredoxin3 can acutely increase their activities and thermogenic respiration.

## 4. The Role of Nrf2 in Energy Expenditure

In addition to the defensive roles against oxidative stress, Nrf2 is a significant player in energy metabolism [73,74]. To show Nrf2 effects in energy metabolism and obesity, Nrf2 can be pharmacologically regulated by its activators or inhibitors. Recently, dietary phytochemicals and small molecules targeting Nrf2 have been extensively reviewed elsewhere [75,76,77,78]. Genetic loss of Nrf2 or Keap1 can lead to constitutive inhibition or activation of Nrf2, respectively. Increased body weight and oxidative stress by high fat diet feeding can be prevented by activating Nrf2 or ablating Nrf2 [17,79,80]. However, the published data on the roles of Nrf2 are not consistent as shown below. In this section, we summarize the current research on the role of Nrf2 inhibition and activation in obesity and energy expenditure.

### 4.1. Nrf2 Inhibition Increases Thermogenesis and Energy Expenditure

Recently, it was described that Nrf2 plays a role in controlling adipogenesis [73,81,82,83,84,85,86]. Several studies have shown that mice lacking Nrf2 can be protected from high fat diet induced obesity despite similar food intake [81,82,87,88]. It appears that anti-obese effects shown in Nrf2 KO mice are due to increased energy expenditure by increasing their metabolic rates. Experiments done by Meakin et al. [89] showed that white adipose tissue mass accumulation was less apparent in Nrf2 KO mice after 8–10 weeks of high fat diet feeding and that Nrf2 KO mice exhibited higher oxygen consumption. Similarly, Schneider et al. [17] provided evidence that Nrf2 deficient mice displayed mitigation of HFD-induced weight gain and that this resistance to diet induced obesity was associated with 20–30% increase in energy expenditure. Despite increased energy metabolism, both wild type and Nrf2 KO mice showed similar food intake and RER, suggesting that these mice did not shift their energy source. Increased oxygen consumption and Ucp1 expression were observed in adipose tissues of Nrf2 deficient mice. In addition, they found that treatment with an antioxidant downregulated Ucp1 expression in Nrf2 deficient mouse embryonic fibroblasts [17]. These findings suggest that increased oxidative stress due to Nrf2 deletion might be responsible for increased cellular respiration and Ucp1 expression, resulting in change of energetics. Collectively, Nrf2 knockout (KO) mice often display a leaner phenotype than wild type mice (Table 1).

In one study using adipocyte specific Nrf2 knockout (ANKO) mice, levels of Ucp1 were found to be almost 75% higher in iWAT but four times lower in eWAT of ANKO mice without showing significant changes in weight gain or energy expenditure [92]. These data imply that the increase of Ucp1 expression in iWAT may not be enough to drive an increase in systemic energy expenditure observed in whole body Nrf2 KO mice. In addition, other studies showed that the absence of Nrf2 did not prevent HFD-induced obesity [90,91]. The controversial outcomes on the role of Nrf2 inhibition in HFD-induced weight gains could be the results of differences in genetic background, sources of the diet, age of the mice on the HFD diets, or diet composition. Nevertheless, these observations point out that the Nrf2 pathway might be an effective means to increase energy expenditure and treat obesity.

### 4.2. Nrf2 Activation Stimulates Energy Metabolism and Prevents Obesity

Effects of Nrf2 activation on metabolic disease including obesity have been investigated using Nrf2 chemical activators [16,79,80,93]. Pharmacological Nrf2 activators such as oltipraz, sulforaphane, curcumin, and 1-(2-cyano-3, 12-dioxooleana-1,9(11)-dien-28-oyl) imidazole(CDDO-Im) are known to induce the expression of Nrf2 both in vitro and in vivo. Effects of Nrf2 activators on obesity were first reported by Shin et al. [14]. The authors showed that CDDO-Im prevented body weight gain upon high fat diet feeding. However, this effect on weight gain was completely lost in Nrf2 KO mice. Moreover, repeated treatment of CDDO-Im increased oxygen consumption and energy expenditure, suggesting that Nrf2 activation by CDDO-Im could induce energy metabolism. Subsequently, several natural and synthetic Nrf2 activators have been found to be effective against obesity. Sulforaphane, an isothiocyanate derived from cruciferous vegetable, is one of the most potent natural Nrf2 inducers. Nagata et al. [15] investigated effects of glucoraphanin, a stable precursor of sulforaphane, in obese mice and found that oral administration of glucoraphanin can significantly decrease weight gains and increased energy expenditure and Ucp1 expression in HFD-fed mice. They further showed that effects of glucoraphanin on weight-gain, whole body energy expenditure, and protein expression of Ucp1 in WAT were abolished in Nrf2 KO mice. Thus, other chemicals (i.e., oltipraz, sesamol, and curcumin) that can activate Nrf2 are presumably good candidates for preventing obesity and improving metabolic diseases possibly through Nrf2 activation [16,79,80,93] (Table 2).

Nrf2 activation can be managed by genetic disruption of the Keap1 gene, a Nrf2 negative regulator [74,83,84,90]. Uruno et al. [18] showed that enhancing Nrf2 signaling by employing hypomorphic allele of Keap1 (Keap1 KD) in mice allows graded expression of Keap1. They also showed that genetic activation of Nrf2 signaling prevented weight gain on normal and high fat diet feeding. Furthermore, the reduction of body weight gain in Keap1 KD was dependent on Nrf2 under both standard diet and high calorie diet-fed conditions. They also demonstrated that energy consumption related gene expression and oxygen consumption in BAT and skeletal muscle of Keap1 KD mice were increased, although tissue weight and locomotor activity were similar. These results imply that changes of energy consumption-related gene expression can lead to increased oxygen consumption and protect from diet-induced obesity and metabolic dysregulations (Table 2). Similarly, Xu et al. [83] reported that HFD-induced body weight gains and lipid accumulation in white adipose tissue were decreased in Keap1-KD mice. However, they also showed that increased Nrf2 activity induced insulin resistance in ob/ob (Lep*^ob/ob^*) genetic background. Zhang et al. [90] showed no significant effect of enhanced Nrf2 activity on body weight gains and insulin sensitivity. Furthermore, Keap1-KD mice, on treatment with HFD for long term (24 weeks), exhibited higher body weight and white adipose tissue mass compared to C57BL/6 mice [84].

The cause of conflicting results may also be due to the differences in the experimental conditions such as diet composition, feeding duration, or genetic background. Considering multiple functions of Keap1, it is also possible that Keap1 plays a role in Nrf2 independent fashion. Future studies are needed to clearly define the role of Keap1-Nrf2 in obesity. Together, even though studies have yielded some conflicting evidence, most of studies have shown that Nrf2 activation can prevent obesity and metabolic diseases.

## 5. How to Resolve Similar Effects of Nrf2 Activation and Inhibition in Preventing Obesity?

Published studies have demonstrated that Nrf2 plays a role in energy metabolism and thermogenesis. However, outcomes on obesity by either Nrf2 activators or inhibitors seem to be confusing. Experiments using Nrf2 KO and Keap1 KD mice as well as pharmacological Nrf2 activator showed contradictory roles of Nrf2 signaling in regulating energy metabolism and thermogenesis [17,18,89,92]. Here, we discuss possible causes for this discrepancy.

How to reconcile this dispute? High ROS levels in Nrf2 KO mice are known to promote Ucp1 activity to increase energy expenditure. Mitochondrial ROS induces heat production in BAT upon meal consumption [65]. Inhibition of oxidation by NAC treatments decreased adrenergic induction of Ucp1 and energy expenditure [70]. Reduction of GSH level and induced oxidized condition increased thermogenic program and drove respiration in brown adipocytes [91]. Based on these findings, it is speculated that high oxidative stress in Nrf2 deficient mice would be responsible for the stimulation of Ucp1 mediated thermogenesis and energy expenditure. Molecular basis for these observations can be explained in that ROS can induce Ucp1 modifications, increase Ucp1 activity, and stimulate energy expenditure during acute adrenergic stimulation.

Alternatively, redox imbalance (oxidative stress) can occur when ROS production exceeds the antioxidant ability. This imbalance is involved in several diseases including metabolic diseases and cancer [94]. Conflicting results of Nrf2 KO mice in obesity are possibly due to high levels of ROS within cells since Nrf2 is a key factor for controlling ROS levels. Production of uncontrollable ROS levels especially in the condition of HFD fed Nrf2 KO obese mice may generate metabolic tissue damage and lead to disruption of whole-body metabolism [17,89,92]. Similarly, in Nrf2 KO mice, sustained high level of ROS under chronic adrenergic stimulation might trigger mitochondrial dysfunction in particularly highly oxidized tissues such as BAT and muscles. Impairments of thermogenic activity in highly stressed Nrf2 KO mice even in ambient temperature would induce BAT/muscle-independent thermogenic mechanisms to maintain body temperature. The BAT/muscle-independent thermogenic mechanisms can be less efficient and consume energy, consequently preventing weight gains upon high calorie diet feeding [3].

Of note, increased Ucp1 expression may also contribute to increased energy expenditure in Nrf2 activated mice. Pharmacological Nrf2 activation via Ucp1 proximal promoter can induce Ucp1 transcription. This plays a part in adrenergic activation induced energy expenditure [39,61]. Similarly, glucoraphanin administrated mice exhibited elevated Ucp1 expression and enhanced energy expenditure [15]. These results are consistent with phenotypes shown in Keap1 haplo-insufficient mice [18]. Therefore, these data suggest that increased Ucp1 activity by sustained ROS in Nrf2 KO mice and induced Ucp1 expression in Nrf2 gain-of-function mice both converge into increase of energy expenditure and prevention of weight gain.

## 6. Nrf2 Activation as Strategies to Enhance Energy Expenditure in Obese Conditions

**Figure 2:**
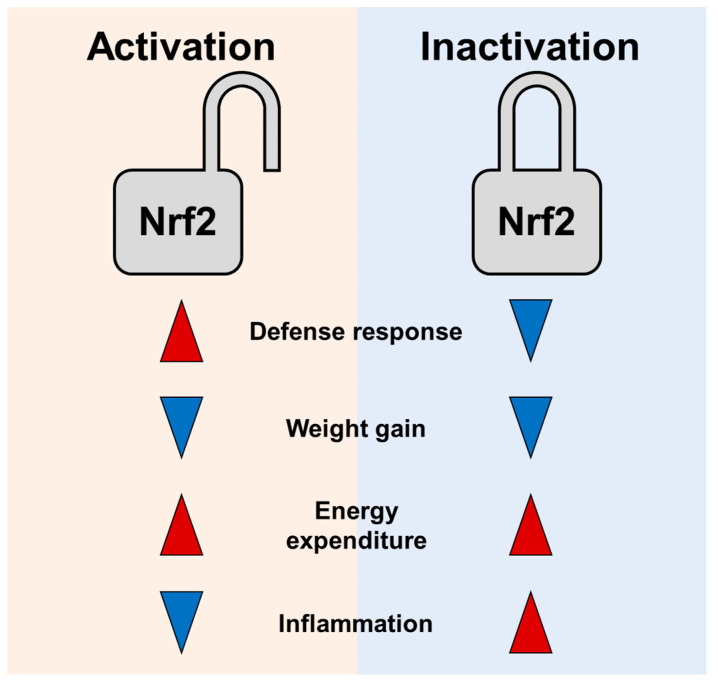
Comparative effects of Nrf2 activation and Nrf2 inhibition on defense responses, weight reduction, and increases of energy expenditure. Schematic diagram shows effects of Nrf2—gain and loss—of function on energy expenditure and weight reduction. Various cellular stresses can activate Nrf2 to provide defense mechanisms in antioxidation and detoxification. Nrf2 inhibition increases energy expenditure and decreases weight gain in a high calorie diet feeding. However, Nrf2 inhibition potentially fails to protect cells from oxidative/electrophilic stress and inflammatory insults. By contrast, chemical Nrf2 activators can stimulate mitochondrial biogenesis in tissues while Nrf2 inhibition impairs mitochondrial biogenesis and energy expenditure.

The primary function of Nrf2 is to provide antioxidative and cytoprotective roles [95]. Beyond cytoprotection, Nrf2 has potential to combat obesity and type 2 diabetes [96]. Nrf2 and ROS can regulate each other. Both display physiological responses, causing Nrf2 to have roles in energy metabolism in a context-dependent manner. Nrf2 suppression or activation exhibits similar anti-obese effects upon HFD feeding. However, as discussed above, unchecked high ROS levels and inflammatory insults in the Nrf2 KO mice are likely to exhibit deleterious physiological responses [97]. Thus, lean phenotype of HFD fed Nrf2 KO mice might be metabolically unhealthy. In addition, chemical Nrf2 activators can stimulate mitochondrial biogenesis in muscle, brain, kidney, and heart [98,99]. By contrast, Nrf2 inhibition by siRNA or Nrf2 knockouts impairs mitochondrial biogenesis and suppresses Pgc-1a, indicating that Nrf2 activation compared to Nrf2 inhibition is more effective in improving energy metabolism [99]. As Nrf2 is shown to negatively control inflammation, Nrf2 inhibition further raises concerns of inflamed status [100]. Therefore, Nrf2 activation might be a better strategy to control ROS levels, metabolism, and energy expenditure than Nrf2 inhibition (Figure 2).

It is tempting to envision that Nrf2 activation might be a safer way than Nrf2 inhibition to defend cells against oxidative stress and increase energy expenditure in obese conditions. However, this activation strategy to induce energy expenditure may also require caution. ROS are signaling molecules in various physiological processes. Consequences of ROS production are contradictory depending on ROS levels [94]. In line with this speculation, treatments with high doses of activators or genetic activation may exhibit unexpected physiological outcomes due to altered ROS levels or Nrf2 activities. Mild Nrf2 activation is likely to extend life span in multiple organisms [101], whereas hyperactivation confers a short-life span. In flies, one copy deletion of Keap1 gene increased life span and protected against stress while complete deletion was lethal during development [102,103,104]. Similarly, moderate activation of Nrf2 by a chemical RU486 increased life span while persistent Nrf2 overexpression in flies or high-copy transgenic SKN-1 in nematodes reduced longevity [103,105]. In rodents, sustained Nrf2 activation by Keap1 deficiency in hematopoietic stem cells caused stem cell exhaustion [106]. Furthermore, Nrf2 activators such as xenobiotics and phytochemicals at high concentrations displayed cellular cytotoxicity by forming DNA, protein, and lipid adducts [34,36].

In addition, Nrf2 activity in certain diseases should be considered. Nrf2 has a role in activating cellular antioxidant response. In addition, it acts as a major regulator of cell survival. Thus, activation of Nrf2-mediated defense can protect against various diseases including cancers [107]. A variety of natural and synthetic compounds that can increase the activity of Nrf2 have been tested for treating diseases. However, studies have demonstrated that activation of Nrf2 can not only promote cell survival by eliminating ROS in normal cells, but can also support cancer cells to survive better by protecting them from oxidative stress, chemotherapeutic reagents, and radiotherapy [108,109]. Indeed, some Nrf2 activating phytochemicals can facilitate cancer cell growth through reduction of ROS levels [39]. Conversely, Nrf2 suppression in cancer cells can restore sensitivity of cancer cells to chemotherapy [110]. Treatment with Nrf2 inhibitor (i.e., apigenin or luteolin) and doxorubicin has shown synergistic anti-tumor effect [111,112,113]. This phenomenon makes it necessary to better understand Nrf2 signaling for treating certain diseases. Taken together, temporal and spatial Nrf2 activation along with nutritional status and physical exercise should be considered for thermogenic responses and energy expenditure. Beneficial effects of temporal, spatial, and degrees of Nrf2 activation associated with energy metabolism and obesity should be further investigated.

## 7. Conclusions

Recent studies have revealed that various chemicals including phytochemicals, synthetic small molecules, or metabolic intermediates can increase energy expenditure. Significant numbers of these small molecules have been indicated or suggested as Nrf2 activators. Given that obesity is associated with high levels of ROS and oxidative stress, these Nrf2 activators can be applied particularly to stressful conditions to remove oxidative stress, induce thermogenesis, and enhance energy expenditure. Therefore, the utility of currently available Nrf2 activators such as drugs and diet supplementation should be further investigated to stimulate energy expenditure in humans, especially under genetic and environmental metabolic dysregulations.

## Figures and Tables

**Table 1:** The effects of Nrf2 inhibition in energy metabolism.

Mouse	Background	Diet (Start/Duration)	Body Mass	IR	Oxidative Stress	Inflammation	EE (Ucp1)	Ref.
Nrf2 KO	C57BL6;129SV	HFD (3–4 w/12 w)	↓					[81]
Nrf2 KO	C57BL6	HFD (9–10 w/180 d)	↓	↓				[87]
Nrf2 KO	C57BL6	HFWD (12 w/12 w)	↔	↔	↓ (GSH in liver)			[90]
Nrf2 KO	ob/ob	SD (4 w/11 w)	↓	↑		↓ (WAT)		[82]
Nrf2 KO	C57BL6	HFD (8 w/4 w)	↔		↑ (MDA in liver)			[91]
Nrf2 KO	C57BL6	HFD (8–10 w/16–20 w)	↓	↑	↑ (GSSG, MDA in liver)	↑ (liver)	↑	[89]
Nrf2 KO	C57BL6	HFD (12 w/6 w)	↓	↑	↓ (GSH/GSSG in WAT)		↑ (↔RER) (↑in WAT)	[17]
Adipo-Nrf2 KO	C57BL6	HFD (6 w/14 w)	↓				↓ RER in SD	[88]
Adipo-Nrf2 KO	Albino C57BL6	HFD (8 w/170 d)	↔	↑		↔	↔ (↑iWAT ↓eWAT)	[92]

KO, knockout; ↑, increased; ↓, decreased; ↔; no change; w, week; d, day; IR, insulin resistance; SD, standard diet; HFWD, high fat Western diet; HFD, high fat diet; ob/ob, leptin deficient obese mice; GSH, reduced glutathione (reduced); GSSG, glutathione disulfide (oxidized); MDA, malondialdehyde; RER, respiratory exchange ratio; EE, energy expenditure; eWAT, epididymal white adipose tissue; iWAT, inguinal white adipose tissue; WAT, white adipose tissue; BAT, brown adipose tissue.

**Table 2:** The effects of Nrf2 activators in energy metabolism.

Treatment /Mouse	Background	Diet (Start/Duration)	Body Mass	IR	Oxidative Stress	Inflammation	EE (Ucp1)	Ref.
Pharmacological activation								
CDDO-Im	C57BL6	HFD (6–7 w/95 d)	↓				↑	[14]
Glucoraphanin	C57BL6	HFD (8 w/14 w)	↓	↓		↓ (liver, eWAT)	↑ (↑WAT, ↔BAT)	[15]
Oltipraz	C57BL6	HFD (5 w/28 w)	↓	↓	↑ (GSH/GSSG)	↓ (eWAT)		[79]
Curcumin	C57BL6	HFD (9 w/18 w)	↔	↓	↓ (muscle) ↔ (adipose and liver)			[80]
Curcumin	C57BL6	HFD (3–5 w/15 w)	↓	↓		↓ (liver, WAT)		[93]
Curcumin	ob/ob	SD (8–10 w/14–18 w)	↓	↓		↓ (liver, WAT)		[93]
Sesamol	C57BL6	HFD (8 w/12 w)	↓	↓		↓ (iWAT)	↑ (↑iWAT)	[16]
Genetic activation								
Keap1 KD	C57BL6	HFD (9 w/36 d)	↓					[83]
Keap1 KD	ob/ob	SD (4 w/8 w)	↔	↑				[83]
Keap1 KD	C57BL6	HFWD (12 w/12 w)	↔	↔				[90]
Keap1 KD	C57BL6	HFD (3 w/24 w)	↑	↑		↑ (liver, WAT)	↑	[84]
Keap1 ^flox/−^	ICR	SD/HCD (4 w/12 w)	↓	↓			↑ (↔BAT)	[18]

↑, increased; ↓, decreased; ↔; no change; w, week; d, day; IR, insulin resistance; HFD, high fat diet; HCD, high carbohydrate diet; HFWD, high fat western diet; SD, standard diet; CDDO-Im, 1-(2-cyano-3, 12-dioxooleana-1,9(11)-dien-28-oyl) imidazole; ob/ob, leptin deficient obese mice; Keap1, Kelch-like ECH-associated protein 1; KD, knockdown; Keap1 ^flox/−^, Keap1 gene hypomorphic knockdown; GSH, reduced glutathione (reduced); GSSG, glutathione disulfide (oxidized); eWAT, epididymal white adipose tissue; iWAT, inguinal white adipose tissue; BAT, brown adipose tissue.
